# Exploring Factors Influencing Motivation and Success in Teaching Patients Pessary Self-Management: A Qualitative Study

**DOI:** 10.1007/s00192-025-06118-w

**Published:** 2025-03-20

**Authors:** Renée Börger, Evy Maria Bernadette Paulussen, Josephine Eissing, Marlies Yvette Bongers, Dorothea Maria Koppes, Mirjam Weemhoff

**Affiliations:** 1 Department of Obstetrics and Gynaecology, Zuyderland Medical Centre, Heerlen, The Netherlands; 2 Department of Obstetrics and Gynaecology, Jeroen Bosch Medical Centre, ‘s-Hertogenbosch, The Netherlands; 3 Faculty of Health, Medicine & Life Sciences, Maastricht University, Maastricht, The Netherlands; 4 GROW, School for Oncology and Developmental Biology, Maastricht University Medical Centre, Maastricht, The Netherlands

**Keywords:** Conservative treatment, Pelvic organ prolapse, Pessary, Qualitative study, Self-management

## Abstract

**Introduction and Hypothesis:**

Pessary self-management (PSM) may increase patient autonomy and minimize the number of doctor consultations. However, little is known about the implementation and patient perception of PSM. This study aims to explore patients’ experiences, opinions, and needs regarding PSM and how this was taught. Furthermore, to develop a standardized instruction manual.

**Methods:**

A qualitative study with semi-structured interviews was conducted among patients with pessary therapy for pelvic organ prolapse (POP). A preliminary framework was conducted for thematic content analysis. Three main themes were described: initial opinion, motivation and suggestions for healthcare workers and other patients regarding PSM.

**Results:**

Seventeen patients were interviewed of which 11 performed PSM, five women refused PSM, and one woman was not able to perform PSM. The initial opinion of patients varied. Half of patients were confident in succeeding to perform PSM, and in the other half, nervousness and lack of confidence in being able to perform PSM predominated. Autonomy, hygiene and fewer doctor consultations were named as the main motivating factors to perform PSM. Reasons for not performing PSM were anxiety and existing comorbidities. Patients were generally satisfied with the way PSM was explained and named videos and information leaflets as tips.

**Conclusions:**

This explorative qualitative study revealed the motivators and barriers to learning PSM. Our findings highlight the importance of addressing patients’ concerns and providing adequate information. In doing so, it is important to create a supportive environment to increase patient engagement and confidence in managing their condition through PSM. These results can help to successfully implement PSM in future healthcare.

## Introduction

Pelvic organ prolapse (POP) affects up to 40% of women aged 45 years and older, often presenting with symptoms such as sensation of a vaginal bulging, pain and incontinence [1]. POP can be managed through either pessary use or surgical intervention, depending on the type of prolapse and patient preferences. Approximately half of the patients with POP opt for surgery, while the other half opt for pessary therapy [2]. In traditional clinical practice, a pessary is removed, cleaned and reinserted two to three times a year, generally performed by a general practitioner or gynaecologist. However, many patients experience these routine check-ups as burdensome [3]. Moreover, pessary therapy with clinical management is frequently associated with side effects such as vaginal discharge, blood loss and ulceration [4].

Teaching women to perform self-management offers several advantages: it enables them to anticipate and decrease side effects by, for example, removing the pessary overnight or once a month [5, 6]. Furthermore, it enhances patient autonomy, and it may reduce the number of doctor consultations in the long term [5, 7]. Previous research, in which pessary self-management (PSM) is taught to patients who were willing to do so, showed success rates up to 73% for treatment continuation at 6 months [5]. A recent qualitative study with interviews on women’s attitude towards PSM identified several benefits and barriers to learning PSM. Motivators for PSM included healthcare provider recommendations and ease of care. Examples of benefits included increased autonomy and avoidance of complications. In contrast, barriers to PSM included structural and psychological challenges, as well as a lack of knowledge and time [3]. This suggests that there is significant potential for improvement in teaching PSM in clinical practice. Furthermore, little is known in the existing literature regarding the implementation and patient perspectives on learning PSM.

The aim of this study was to enhance understanding in women’s perspectives on PSM and to explore how training in PSM can be effectively offered in future healthcare settings. This qualitative study collected patients’ opinions, experiences, and needs regarding training in pessary self-care, to promote PSM and to implement PSM in future healthcare practice.

## Materials and Methods

The Medical Ethical Research Committee exempted the need for ethical review (METCZ Z2021035), as it was not subject to the WMO.

A qualitative study with semi-structured interviews was conducted between September 2021 and October 2023 to evaluate patients’ opinions and experiences regarding the process of training in pessary self-management.

Patients with POP who received a ring pessary for PSM (with or without support, with or without knob) at the participating medical centre, were eligible for this study. Patients were excluded if they were under the age of 18, had insufficient knowledge of the Dutch language or were not able to understand the study information or to perform self-management due to physical impairment.

The instruction on self-management was provided by the gynaecologist that fitted the pessary. This could be after the fitting procedure or in a follow-up appointment when the pessary therapy was evaluated. Patients were advised to remove, clean and replace the pessary at least twice a year. They were informed that there is no standard recommendation regarding the frequency of changing the pessary. Patients were allowed to determine the frequency based on their own preferences. Subsequently, patients were asked if they would like to participate in the study. If patients were interested in participating, they were contacted by phone afterwards for an explanation about the design and scheduling an interview. All women signed their informed consent.

Three groups were included in this study: patients who had been informed about self-management but declined (group 1), patients who had been informed and instructed about PSM, tried it, but did not succeed (group 2), and patients who had been informed and instructed about PSM and continued to perform it (group 3).

Semi-structured interviews were conducted by two authors (EP, JE) and took place by telephone. Each interview consisted of a short introduction at which baseline characteristics were collected followed by predetermined, open explorative questions based on a specific topic list (A1). Interviews were conducted until saturation was reached.

All interviews were audio-recorded and transcribed verbatim anonymously using transcription software (Amberscript) after which they were also manually verified (RB, EP, JE). A preliminary framework based on interview questions was used for thematic content analysis (deductive). While encoding interviews, this framework was adapted by adding and changing themes (inductive) (A2). To guarantee intercoder reliability, 20% of the interviews were encoded independently by three different researchers (DK, EP, JE). Systematic content analysis was performed using the qualitative software package Atlas-ti (version 23.2.1). The analysis and coding were discussed until consensus was reached. Each theme was ranked to show which facilitating factors were most desirable in developing the optimal standardized instruction for PSM.

## Results

Seventeen women from the Zuyderland medical centre who had received a pessary were enrolled in this qualitative study. Saturation was reached after 17 interviews. The interviews lasted 6 to 17 minutes each. Among the participants, 11 women performed PSM, five women refused PSM, and one woman received instructions, but was unable to perform PSM. The participants ranged in age from 48 to 79 years. The baseline characteristics are summarized in Table 1.

**Table 1:** Baseline characteristics

	PSM (*n* = 11)	No PSM (*n* = 6)
Mean age (years)	60	71
Level of education		
Low	4	0
Middle	0	5
High	7	1
Menopausal status		
Pre	2	0
Post	9	6
Sexually active		
Yes	11	2
No		2
Unknown		2

*PSM* pessary self-management, PSM = group 3, no PSM = group 1 and 2

Our findings in the perspectives of patients regarding PSM could be divided into three main themes: (1) initial opinion about PSM, (2) motivation to opt for (or not to opt for) PSM, (3) suggestions/tips/wishes for healthcare workers and other patients on learning PSM.

### Initial Opinion on PSM

The initial opinions of patients regarding self-management varied. Some women expressed positive feelings, such as confidence in their ability to succeed at PSM. Women who were initially optimistic about PSM, were often the ones most eager to learn and succeed in performing PSM. One woman, who initially had a moderately positive and apprehensive opinion, ultimately learned self-management after receiving good explanation and trust.

Patients described managing their own pessary as a way to better understand your own body, practical and important. As one patient explained:

“It’s just a matter of holding it under the tap, rinsing it with water, and it’s good to go back in. I compare it to contact lenses. I take them out at night and put them back in in the morning. With the ring, I do that once a week.”

### Motivation to Opt for Pessary Self-Management

The most commonly voiced motivators for performing PSM were pragmatic, hygiene and autonomy. Patients described removing and cleaning their pessary two or three times a year as unhygienic. Patients felt more autonomy, when they were able to remove their pessary before sexual intercourse or when they could change their pessary when they wanted. Furthermore, patients expressed appreciation for the fact that no one needed to examine their genitals, and they did not have to worry about encountering different healthcare providers repeatedly (Table 2).

**Table 2:** Motivation to opt for pessary self-management and sub-themes with quotes

Sub-theme	Quotes
Autonomy	“I’m fine with doing it myself, so I can maintain control.”
	“You don’t have to go to the doctor every time; instead, you can clean it yourself whenever it suits you. You have control over it yourself.”
Pragmatic/practical	“If you lose it or find that it doesn’t fit well, you can adjust it yourself without having to wait for the gynaecologist.”
	“Especially the fact that I don’t have to return as often or visit the general practitioner for this anymore.”
Hygiene	“And then I thought to myself, once every 6 months, that’s really quite long.”
	“Well, how do you say it, in those 5 weeks when you’ve had intercourse, then it smells, I did want to wash it then, but I didn’t dare to take that thing out yet to clean it properly. If I had known how easy it was to replace it, I would have taken it out immediately, cleaned it, and put it back.”

### Motivation Not to Opt for PSM

One of the most important reasons for not wanting to learn self-management was comorbidity. Comorbidities that made self-management difficult included arthritis or rheumatism in the hands. A patient commented: “I have arthritis in my fingers, so I have great difficulty in compressing things, so it seems to me that insertion of the pessary would be very difficult.”

Another important reason was that patients were afraid of causing damage or incorrectly repositioning the pessary. “I am afraid that it might not be positioned correctly and if you had a tampon that was not positioned properly, it caused pain and then I might not be able to remove it myself.” Patients were asked whether shame was part of the reason for not wanting to engage in PSM, but they refuted that. Particularly, tension was then added as the reason. “No, no. Not really shame, just a lot of tension.”

### Suggestions Regarding PSM

In these suggestions a distinction was made between suggestions for healthcare workers (Table 3) and suggestions for other patients (Table 4). Women reported emphasizing that nothing can go wrong when replacing the pessary, as one of the most important recommendations. In addition, visual information (i.e. videos or folders) was desired when explaining PSM. Two patients named help from their partner as a tip for PSM. The recommendations were divided into three subcategories: attitude, informative and training.

**Table 3:** Suggestions regarding pessary self-management for healthcare workers

Subtheme	Quotes
Attitude	“No, I really felt like she gave me that confidence, you can do that yourself too.”
	“Yes, definitely, they should have patience, I think, because it’s for most people the first time, taking it out and putting it back in. You need to calm the patient.”
Informative	“In hindsight, it might have been handy if I had received a small instruction with something visual indeed, so that I could have looked back at it again at home.”
	“I think a short video, just a brief clip, to see how it’s done, and then you see it immediately.”
	“Because she said: you can never insert it incorrectly, so you don’t need to be afraid of it.”
Training	“To do it for the first time under the guidance of a physician is indeed the best approach.”
	“I would rather try it myself, perhaps with a nurse or a doctor, so that I know I’m doing it correctly.”

**Table 4:** Suggestions regarding pessary self-management for other patients

Subtheme	Quotes
Attitude	“I would just say, try it yourself. You need to stay calm.”
Informative	“Potentially, a personal story from a fellow sufferer.”
	“I think the first thing would be that website about the pelvic floor that the gynaecologist showed me. If you read that information thoroughly, you are probably already very well informed.”
Training	“You actually have to learn it by doing it a lot.”
	“To do it for the first time under the guidance of a physician is indeed the best.”

## Discussion

In this qualitative study we explored women’s opinions, experiences and motivation regarding pessary self-management to promote and implement PSM in future healthcare. Most women were positive and confident that PSM would succeed. However, some women expressed initial nervousness and a lack of confidence, highlighting potential apprehension among certain individuals. Key motivators for performing PSM included increased autonomy, hygiene and fewer doctor consultations. Conversely, anxiety and comorbidities were cited as reasons for refusing PSM. Concerns about causing damage or incorrectly repositioning the pessary, indicating the need for reassurance and education on the safety and efficacy of PSM. Patients reported satisfaction with the explanations provided for PSM and found resources such as videos, information leaflets and websites particularly helpful. They appreciated the opportunity to ask questions and practice PSM techniques under guidance. Additionally, patients offered advice to others, encouraging them to “just try it” and “have faith in yourself”, highlighting the importance of confidence and perseverance in successfully implementing PSM.

In accordance with the present results, the study of Stairs et al. emphasized the importance of patient education on benefits of PSM, alongside efforts to normalize patient engagement in PSM. Additionally, they reported autonomy and hygiene as motivators to perform PSM. This was in line with our study. Women named comorbidities as a barrier to perform PSM. This result also agreed with other research which found women being more likely to perform PSM if they had fewer comorbidities. Further, higher level of education was associated with women being more likely to perform PSM [8]. Holubyeva et al. found that patients with characteristics of younger age, being premenopausal and having a lower stage of POP were more likely for PSM. Consistent with the literature, women in our study stated that they prefer removing their pessary before sexual activity [9]. Dwyer et al. found that 70% of women removed their pessary usually or always before sexual intercourse [8]. In the women’s experiences, specialized nurses played a primary role in support during learning and practicing PSM [10]. Women also reported valuing the rest and guidance provided during their practice session. Unexpectedly, women did not suggest having a nurse’s telephone number for questions or help with pessary replacement, contrary to our expectations.

This study is one of the few qualitative studies that looked at the opinions and experiences regarding training of pessary self-management. The comparability of these results was therefore limited. However, this qualitative study design allowed us to hear patient opinions about training PSM and gave the ability to improve interventions with patient centred data. The suggestions made in this study were relevant and were implemented in a new patient centred standardized manner to teach self-management to women with a ring pessary (with or without support, with or without knob).

A strength of this study was that women of all ages and education levels were included. This is a good representation of the population. Furthermore, a search of the literature revealed no other studies that interviewed both patient groups, women who were willing to perform PSM and women who preferred CM. A limitation of this study was that the interviews were conducted over the phone and in telephonic interviews nonverbal communication is lacking. However, this study started during COVID-19 and for uniformity, the interviews were also conducted by telephone thereafter.

In conclusion, this explorative qualitative study showed which motivators and barriers there were to learn PSM. Our findings highlighted the importance of addressing patients’ concerns and providing adequate information and resources. Thereby it is important to foster a supportive environment to enhance patient engagement and confidence in managing their condition through PSM. These results can help to successfully implement PSM in future healthcare. Future qualitative research could focus on differences in quality of life between women with PSM and CM.

## Data Availability

The data that support the findings of this study are available from the corresponding author, [RB], upon reasonable request.

## Appendices

### Appendix 1: Interview Scripts

#### Demographics

What is your age?

What is your highest level of education completed?

Are you still menstruating?

If no, when was your last menstrual period?

Have you ever used a pessary before?

Do you have a partner?

Are you sexually active?

### GROUP 1 (Rejects Self-Management)

#### Opening/first questions

Could you indicate how you feel about self-management?

How did the doctor introduce the concept of self-management? Prompts:

Was it discussed over the phone or face to face?

What did the doctor specifically explain about self-management?

Did he or she use a picture or drawing, or was the explanation only verbal?

Do you have any questions about self-management that haven’t been answered before?

#### Main Questions about Motives/Reasons

You mentioned that you do not want to learn self-management, is that correct? Could you explain why or what reasons you had for preferring not to learn self-management?

How confident were you in your ability to insert and remove the pessary yourself?

Could you explain why you thought it would or would not succeed? Prompts for not succeeding: fear, shame, physical reasons, etc.

#### Main Questions about Desires/Needs

What did you think of the way the doctor introduced self-management?

Would you have preferred the doctor’s explanation in a different way? If yes, how?

Looking back at the information from your doctor in which he/she offered you self-management; what aspects do you think should be addressed to decide about whether or not to learn self-management?

If you had received that information, to what extent do you think you would have considered trying self-management? And why?

### GROUP 2 (Received Self-Management Instruction but did not Continue)

#### Introduction Questions

Could you indicate how you feel about self-management?

How did the doctor introduce the concept of self-management? Prompts:

Was it discussed over the phone or face to face?

What did the doctor specifically explain about self-management?

Did he or she use a picture or drawing, or was the explanation only verbal?

Do you have any questions about self-management that haven’t been answered before?

#### Main Questions about Motives/Reasons

How confident were you that self-management would succeed?

What reasons were there why you thought it would or would not succeed? Prompts: physical reasons? Fear? Shame?

What motivated you the most to learn self-management?

If multiple answers: what was the most decisive factor? Prompt: feeling of control over own body/care, hygiene, financial reasons.

#### Main Questions about Desires/Needs

After the doctor gave you instructions/taught you how to insert and remove the pessary yourself, you tried to do it at home. Could you tell me how you experienced self-management? (Let the patient share how they have experienced the past weeks.)

What reasons were most important for you to stop self-management? Prompts: it didn’t work (couldn’t reach it well, didn’t know the right position), fear, discomfort, uncertainty that it was not positioned correctly.

What things do you think a ‘new patient’ should know when deciding between self-management or not self-management?

How do you think a ‘new patient’ should receive the explanation and instructions about self-management? What should that explanation consist of? Prompt: brochure, video, phone call, etc.

Did you feel the need to take the explanation home to review or re-read it?

Are there things that could have helped you to continue self-management? Prompt: a certain form of support such as calling, a video, brochure, FAQ, etc.

### GROUP 3 (Received Self-Management Instructions and Continued)

#### Introduction Questions

Could you indicate how you feel about self-management?

How did the doctor introduce the concept of self-management? Prompts:

Was it discussed over the phone or face to face?

What did the doctor specifically explain about self-management?

Did he or she use a picture or drawing, or was the explanation only verbal?

Do you have any questions about self-management that haven’t been answered before?

#### Main Questions about Motives/Reasons

How confident were you that self-management would succeed?

What reasons were there for why you thought it would or would not succeed? Prompts: physical reasons? Fear? Shame?

What motivated you the most to learn self-management?

If multiple answers: what was the most decisive factor? Prompt: feeling of control over own body/care, hygiene, financial reasons.

#### Main Questions about Desires/Needs

After the doctor gave you instructions/taught you how to insert and remove the pessary yourself, you tried to do it at home. Could you tell me how you experienced self-management? (Let the patient share how they have experienced the past weeks.)

What reasons were most important for you to continue self-management?

What things do you think a ‘new patient’ should know when deciding between self-management or not self-management?

How do you think a ‘new patient’ should receive the explanation and instructions about self-management? What should that explanation consist of? Prompt: brochure, video, phone call, etc.

Did you feel the need to take the explanation home to review or re-read it?

Are there things that could have helped you to continue self-management? Prompt: a certain form of support such as calling, a video, brochure, FAQ, etc.

## Appendix 2: Code Tree

1. Age
2. Level of Education
3. Last Menstruation
  A3.1 Premenopausal
  A3.2 Postmenopausal
4. Previous Pessary Use
  A4.1 Yes
  A4.2 No
5. Partner
6. Sexually active
7. Duration Pessary Therapy/Self-Management
8. Other Patient Information
9. Initial Opinion Towards Pessaries
10. Initial Opinion Towards Self-Management
11. Doctor’s Attitude
12. Explanation
13. Timing Introduction
14. Timing teaching PSM
15. Motivation
16. Treatment by the General Practitioner
17. Suggestions
18. Advice/Tips for Other Patients
19. First Experience
